# Supplementary material for: SARS-CoV-2 infection is associated with higher chance of diabetes remission among Veterans with incident diabetes
Source: PLoS One. 2025 Feb 19;20(2):e0317348. doi: 10.1371/journal.pone.0317348 (PMC11838867; doi:10.1371/journal.pone.0317348)
Supplement: Attachment — Submitted filename: rev resp_121924.docx [file pone.0317348.s002.docx]

19 December 2024

Dear editors:

Thank you for your thoughtful review and for the opportunity to revise this manuscript. We respond to the reviewers’ comments point-by-point below.

Great gratitude,

Luke

Pandora Lucrezia “Luke” Wander, MD, MS, FACP

Associate Professor of Medicine

Adjunct Associate Professor of Epidemiology & Public Health Genetics

University of Washington

Staff Physician

Veterans Affairs Puget Sound Health Care System

Reviewers' comments:

1. In the previous round of reviews, I asked about the importance of having 2 separate diabetes cohorts. The answer given was that the authors believed that a recent SARS-CoV-2 infection might impact healthcare utilization and thereby diagnosis rates. Whereas I understand and agree with this view, I am not sure it answers the question posed. There are 2 diabetes cohorts, one defined by lab values and prescriptions and the other cohort defined by A1C. Each cohort is further stratified by COVID status. The comparison I am querying is between those in cohort 1 who have T2DM and COVID and those in cohort 2 who have T2DM and COVID. Why were these data separated? Why were there separate definitions for T2DM?

Thank you for the opportunity to clarify. As there is no generally accepted ideal EHR-based phenotype for new-onset diabetes, we chose two methods, a “specific” method that would yield a higher positive predictive value and minimize false positive diagnoses, and a “sensitive” method that would be more likely to capture true positive diagnosis but also be more likely to include a higher number of false positive diagnoses. A specific EHR definition such as the one we used for cohort 2, which required two HbA1c values ≥6.5% for inclusion, runs a low risk of including individuals who do not truly have diabetes, but may result in the exclusion of so many individuals in the dataset that generalizability is low. On the other hand, a sensitive EHR definition such as the one we used for cohort 1, which used all available data on ICD10 codes, glucose values, HbA1c values and glucose-lowering medication use, is more generalizable but may result in inclusion of individuals who do not truly have diabetes. To balance these priorities, we chose to examine the association of SARS-CoV-2 with remission in both cohorts. Importantly, all individuals included in the specific (cohort 2) were also included in the more sensitive cohort (cohort 1). To be explicit, the population with COVID and T2D in cohort 2 is a subset of the population with COVID and T2D in cohort 1. Our results demonstrate in general similar point estimates for effect measures, thereby increasing confidence in their validity across broader and more targeted diabetes definitions. We added the following text to the methods (p. 6, line 23): “We defined two different diabetes cohorts with higher sensitivity or higher specificity because no universally agreed upon diabetes phenotype exists based on EHR data. Cohort 1 therefore would be less likely to miss capturing persons with diabetes, while cohort 2 would be less likely to falsely misclassify someone without diabetes as having this condition.”

2. New comment: In the discussion, a section was added on remission by vaccine status. Introductory blurb in the introduction or objective sections would be useful.

We added the following text to the introduction (p. 5, line 2): “We also hypothesized that receipt of more SARS-CoV-2 vaccination doses might be associated with a higher chance of remission.”
